# Why Do Children Who Solve False Belief Tasks Begin to Find True Belief Control Tasks Difficult? A Test of Pragmatic Performance Factors in Theory of Mind Tasks

**DOI:** 10.3389/fpsyg.2021.797246

**Published:** 2022-01-14

**Authors:** Lydia P. Schidelko, Michael Huemer, Lara M. Schröder, Anna S. Lueb, Josef Perner, Hannes Rakoczy

**Affiliations:** ^1^Department of Developmental Psychology, University of Göttingen, Göttingen, Germany; ^2^Centre for Cognitive Neuroscience, University of Salzburg, Salzburg, Austria; ^3^Department of Psychology, University of Salzburg, Salzburg, Austria

**Keywords:** Theory of Mind, pragmatics, true belief, false sign task, knowledge, false belief

## Abstract

The litmus test for the development of a metarepresentational Theory of Mind is the false belief (FB) task in which children have to represent how another agent misrepresents the world. Children typically start mastering this task around age four. Recently, however, a puzzling finding has emerged: Once children master the FB task, they begin to fail true belief (TB) control tasks. Pragmatic accounts assume that the TB task is pragmatically confusing because it poses a trivial academic test question about a rational agent’s perspective; and we do not normally engage in such discourse about subjective mental perspectives unless there is at least the possibility of error or deviance. The lack of such an obvious possibility in the TB task implicates that there might be some hidden perspective difference and thus makes the task confusing. In the present study, we test the pragmatic account by administering to 3- to 6-year-olds (*N* = 88) TB and FB tasks and structurally analogous true and false sign (TS/FS) tasks. The belief and sign tasks are matched in terms of representational and metarepresentational complexity; the crucial difference is that TS tasks do not implicate an alternative non-mental perspective and should thus be less pragmatically confusing than TB tasks. The results show parallel and correlated development in FB and FS tasks, replicate the puzzling performance pattern in TB tasks, but show no trace of this in TS tasks. Taken together, these results speak in favor of the pragmatic performance account.

## Introduction

Theory of Mind (ToM) is the ability to impute mental states, such as beliefs and desires, to oneself and to others (Premack and Woodruff, 1978). The developmental litmus test of ToM are so-called false belief (FB) tasks in which children need to ascribe a mistaken belief to another agent and predict her actions accordingly (Wimmer and Perner, 1983). In the standard change-of-location FB task, the protagonist Maxi puts his chocolate in the blue cupboard and leaves. He does not see that his mother then moves the chocolate to the green cupboard. Children are then asked where Maxi, upon return, will look for his chocolate. Children thus have to represent Maxi’s misrepresentation (chocolate in blue cupboard) of the situation (chocolate really in green cupboard). Decades of research with these explicit verbal tasks consistently show that children typically start to ascribe FB around age 4 (Wellman et al., 2001). Success in the FB task goes along with emerging competence in conceptually related tasks that all require metarepresentation – indicating a major conceptual transition in ontogeny (Perner, 1991; Perner and Roessler, 2012).

Before age 4, children systematically fail FB tasks, but pass parallel true belief (TB) control conditions. The TB condition is structurally like the FB condition with the only difference that Maxi watches his mother relocate the chocolate and thus holds a TB^1^ about the chocolate’s location (Wimmer and Perner, 1983). The test question, like in FB conditions, is variously “Where will Maxi look for his chocolate?” or “Where does Maxi believe his chocolate is?”

The TB condition was devised for younger children who fail the FB condition in order to rule out that FB failure is due to general problems with the narrative structure of the task. Only recently was it administered to a broader age range of children, with puzzling patterns of results. Children who begin to solve the FB task suddenly start to fail the TB task. From age 4 to roughly age 10, then, they systematically answer the TB question incorrectly (predicting that Maxi will erroneously look in the old location; Friedman et al., 2003; Fabricius et al., 2010; Oktay-Gür and Rakoczy, 2017; Rakoczy and Oktay-Gür, 2020; Schidelko et al., 2021).

What do these strange findings mean? One possibility is that they reflect a competence limitation in children’s ToM. Contrary to what findings from FB tasks suggest, these findings may be taken as an indication that children do not really engage in metarepresentation until much later (Fabricius et al., 2010, 2021; Hedger and Fabricius, 2011).

Another possibility is that children’s difficulty with TB tasks merely reflects pragmatic performance, rather than competence limitations. According to a pragmatic task analysis, TB tasks may be difficult for children from age 4 to 10 because they combine several factors that make the target question pragmatically confusing and thus demanding (Rakoczy and Oktay-Gür, 2020; for related proposals regarding the role of pragmatic factors in FB and other ToM tasks, see, e.g., Siegal and Beattie, 1991; Helming et al., 2014, 2016; Westra, 2016).

*First* of all, the TB question is an academic test question. Regular questions are asked because the speaker herself does not know the answer and requests the missing piece of information from the interlocutor (Searle, 1969). Academic test questions, in contrast, have a much more complex intentional and pragmatic structure: The speaker wants to know whether the interlocutor knows the answer that the speaker knows perfectly well herself. This special question format appears to be difficult to understand for young children (Siegal, 1999).

*Second*, the TB question is highly trivial: Here, in the story, is a protagonist, who has all the information needed, and now the question is where he will look for an object. The answer is so obvious and common knowledge that it may be difficult to make sense of the corresponding question even if it is understood as an academic test question: Why would someone want to test whether I know what everyone knows?

*Third*, this may be particularly pronounced in the TB case where children are asked where Maxi thinks his chocolate is, or to predict where he will look for it in a situation where he shares common ground and is not subject to any error. Questions in such a context are pragmatically unnatural (Papafragou et al., 2007): We ask for action prediction or explanation or belief ascription only if there is at least the possibility of error and misrepresentation. The test question “What does he believe?” or “What will he do?” therefore suggests that there ought to be an alternative perspective or misrepresentation involved. Yet, the storyline of the TB task does not provide any obvious possibility for error or misrepresentation; children may thus think that they must have missed something and look for a possible alternative perspective on the scenario.

Previous studies have found preliminary evidence for the importance of the first two factors. When tested in a completely non-verbal version of the TB task that removed any (academic and trivial) question, or in a verbal version in which the triviality of the TB question was made explicit (“I’ll ask you a baby question”), children between ages 4 and 7 showed no problems with the TB question (Rakoczy and Oktay-Gür, 2020, *Exp. 1 and Exp. 5*).

But what about the third factor that the test question evokes wondering about an alternative perspective? Preliminary evidence comes from one recent study that compared FB/TB tasks with an analogous task that involves non-mental representations, the False Photo (FP) task (based on Zaitchik, 1990). In the FP task, structurally matched to the FB task, an object is put into location 1 and a polaroid camera takes a photo of the scene. While the photo develops, the object is then moved to location 2, and children are asked where the object is in the outdated (“false”) photo. Earlier studies revealed that the majority of 3-year-olds failed in both tasks, while the majority of 4-year-olds and older children passed both tasks (with a slightly higher performance in the FB task; Zaitchik, 1990; Leekam and Perner, 1991). Rakoczy and Oktay-Gür (2020, *Exp. 2*) thus used the photo task to explore how asking questions about a rational agent’s action or mental perspective may make the TB task pragmatically complex. Four- to 6-year-old children’s performance in FB/TB tasks was therefore compared with their performance in analogous False/True Photo tasks. In the new true photo (TP) condition – in close analogy to the TB story – the camera took the photo after the object had already been moved to the new location. Holding the first two factors (trivial academic test question) constant and manipulating only the third (in TB, but not in TP, the test question implicates that there may be an alternative mental representation), the TP condition implements a crucial contrast case: a trivial academic test question about a non-mental representation (of the photo). Consistent with previous findings, 4- to 6-year-old children succeeded in both “false” conditions (FB and FP) whereas their performance in the “true” conditions was markedly different. They showed the previously noted difficulty in the TB tasks (performing below chance), but not in the TP task (performing above chance) (Rakoczy and Oktay-Gür, 2020, *Exp. 2*). These findings thus provide *prima facie* evidence that it really does matter whether trivial academic test questions implicate an alternative perspective due to the agent’s mental misrepresentation (rather than referring to non-mental representations).

However, the specific contrast used in that study – between FB and FP tasks – makes the findings difficult to interpret. The reason is that the FP task, strictly speaking, does not involve a misrepresentation. From a theoretical point of view, the “false” photo is actually not false but only outdated (it does not falsely depict the scene at time 2, but depicts the scene as it was at time 1; Perner and Leekam, 2008). Empirically, this analysis is corroborated by findings that FB and FP, though both come to be mastered around the same age, dissociate (fail to correlate) in both typical and atypical development (for an overview, see Perner and Leekam, 2008).

A better task that does involve non-mental misrepresentations is the false sign (FS) task. In this task, in structural analogy to the FB task, a sign post in a story scenario indicates a state of affairs (e.g., that an object is in location 1). The object then moves to a new location (location 2), but the signpost is not changed accordingly and therefore becomes a FS (Parkin, 1994). To solve the task, children need to understand that the actual situation is different from how the sign represents it. Importantly, the sign that shows at time 2 that the object at location 2 is at location 1 is not just outdated (like the photo at time 2 showing that the object was earlier in location 1); it is misleading and false. Empirically, this analysis receives support from a number of studies that suggest that FS and FB tasks are related developmentally in ways in which FB and FP are not: Mastery in both tasks does not only emerge around the same age, they are also highly correlated in typical and atypical development (for an overview, see Perner and Leekam, 2008).

The present study thus capitalizes on this, and develops true and false versions of the sign task (TS/FS) as a minimal contrast to FB/TB tasks in order to test more stringently whether it matters for pragmatics whether trivial academic test questions implicate that there may be alternative mental representations or analogously alternative non-mental representations. The general rationale is the following: If indeed there is a major conceptual transition to metarepresentational thinking around the age of 4, the following pattern of results should be found. Performance in different perspective tasks should show parallel trajectories: younger children tend to fail all tasks requiring an understanding of misrepresentation (e.g., FB and FS), whereas older children tend to master all of them. But if a task poses additional task demands, for example pragmatic factors, no such clear parallel pattern is to be expected. More specifically, if indeed the TB tasks pose pragmatic demands that the TS task lacks (since only in the TB task the test question evokes that there could be an alternative mental representation), we should expect divergent performance: older children worsen in TB but not in TS tasks.

We would thus expect, first, positive correlations of performance between FB and FS tasks and negative correlations between FB and TB tasks as documented in previous studies (e.g., Sabbagh et al., 2006; Leekam et al., 2008 for positive correlations of FB and FS tasks, and Oktay-Gür and Rakoczy, 2017 for negative correlations of FB and TB tasks). Second, we would expect dissociations in performance between TB and TS tasks. Children’s performance in the TB task follows the characteristic U-shaped developmental pattern whereas the performance in the TS task will not.

We additionally explored a secondary factor causing wrong answers to the TB question. As in the standard TB task, Maxi watches passively the location change, recent evidence suggests that perhaps children wonder whether Maxi really pays attention and witnesses the location change (Huemer et al., 2019). If children do assume that Maxi did not register the location change, their answer that Maxi will look for the chocolate in its old location would make perfect sense. To reduce any possible ambiguity in this respect Maxi accompanies the location change and we explicitly asked half of the children whether Maxi had seen the location change, as a direct test of whether they have accepted this crucial premise.

## Methods

### Participants

One-hundred-six 3- to 6-year-old German children participated in the study. They were recruited *via* the platform ‘‘KinderSchaffenWissen’’^2^ and from a databank of children whose parents had previously given consent to experimental participation. The final sample consisted of 88 children (46 female, 42 male; range = 36–83 months, *M* = 59.3 months), divided into groups of 3-year-olds (*M* = 42.1 months), 4-year-olds (*M* = 53.9 months), 5-year-olds (*M* = 64.5 months), and 6-year-olds (*M* = 76.7 months), each consisting of 22 children. For more detailed information on participants and exclusion criteria, see Supplementary Material.

### Design

Each child received six test trials: two TB and two FB trials (in blocks) and one FS and one TS trial. The order of FB and TB blocks, and of FS and TS trials was counterbalanced. Whether the *Confirmation-of*-See*ing question* on TB trials was asked or not was varied between participants. The same held true for *Confirmation-of-Change question* on TS trials. For information on the coding procedure, counterbalancing of story plots, number of trials and task protocols, see Supplementary Material.

### Procedure

The study was tested in a moderated online setting *via* a video conferencing platform (mainly *BigBlueButton*). During the test session, the child and a female experimenter communicated *via* audio and video streaming. The experimenter presented the tasks as animated stories *via* shared screen.

#### True and False Belief Task

**FIGURE 1:**
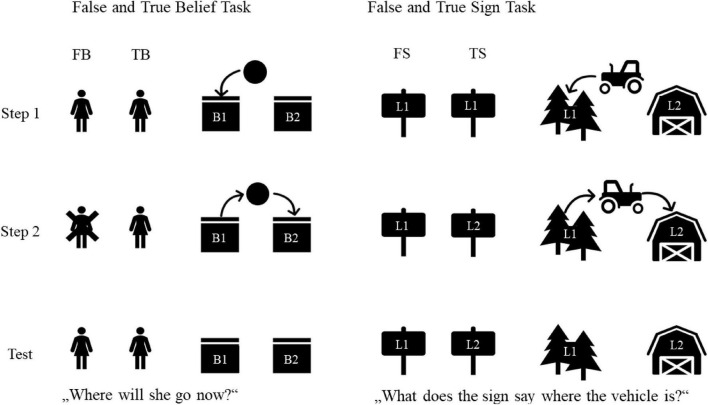
Schematic procedure of False/True Belief and False/True Sign Tasks. Children were classified as passers on a given task if they got two out of two correct on belief tasks and one out of one correct on sign tasks (This makes it easier to pass the sign tasks than the belief tasks. We therefore conducted the same analysis as reported below with the first TB and first FB trial only. These analyses show the same results as reported below, see Supplementary Material.). They were classified as non-passers otherwise.

The change-of-location task (after Wimmer and Perner, 1983; used in, e.g., Perner et al., 2011) was presented as an animated slide show in four parallel story lines (see Figure 1 for task structure). Protagonist 1 placed an object in one of two boxes (B1). Protagonist 2 then transferred the object into the other box (B2) before (TB condition) or after (FB condition) protagonist 1 left the scene. Immediately after protagonist 2 had left the scene, children in the *Confirmation-of*-See*ing question* condition (TB trials only) were asked:

*Confirmation-of*-See*ing question (TB)*: Did [*protagonist 1*] see that? (*Correct answer: yes*)

After that, protagonist 1 returned and children were told that protagonist 1 wanted to have her object now. Then, children were asked the following questions:

*Test question:* Where will [*protagonist 1*] go now?^3^ [*Correct answer: B2 (TB), B1 (FB)*]
*Memory question:* Where did [*protagonist 1*] put the object in the beginning? (*Correct answer: B1*)
*Reality question:* Where is the object now? (*Correct answer: B2*)

#### True and False Sign Task

The sign task (adapted from Parkin, 1994) was presented in two storylines. In a familiarization, children were introduced to the setting and learned that the color of the sign at the crossing indicates the location of the vehicle. In the test trials (see Figure 1 for task structure), the vehicle drove to location 1 (L1) and the sign showing color 1 was placed at the crossing. After a quick stop at L1 the vehicle drove off again stopped briefly at the crossing, either with (TS condition) or without (FS condition) changing the sign to color 2, and then continued to L2. Half the children were then asked the *Confirmation-of-Change question* on TS trials:

*Confirmation-of-Change question* (*TS*): Was the sign changed? (Correct answer: yes)

Once the vehicle had stopped at L2, children were asked further questions:

*Test question*: What does the sign say where the [*vehicle*] is? [*Correct answer: L2 (TS), L1 (FS)*]
*Reality question*: Where is the [*vehicle*] now? (*Correct answer: L2*)
*Memory question*: And where was it right before? (*Correct answer: L1*)

Importantly, the sign was a rectangular colored plate (see Figure 1) without a directional feature (no arrow). This adaption was recently introduced for German-speaking populations to ensure that children need to understand the representational feature of the sign (Schuster et al., 2021).

## Results

### Plan of Analysis

**FIGURE 2:**
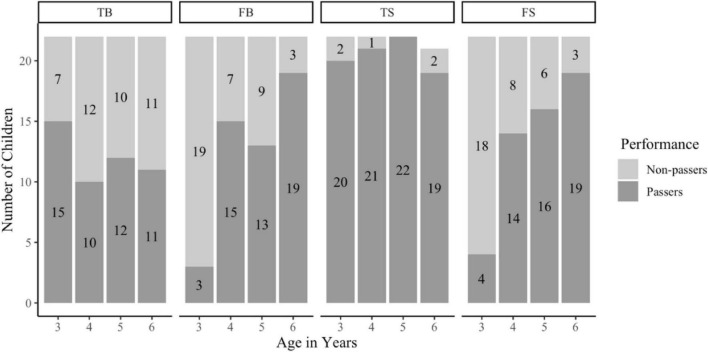
Number of children passing and failing true and false versions of the belief and sign task as a function of age.

The main and novel focus of the present study was on the relation between TB and TS (the former should, while the latter should not show a performance decline after age 4). This focal analysis is only meaningful, however, against the background of two presuppositions: That the patterns of negative correlations between TB and FB and positive correlations between FB and FS performance found in previous studies can be replicated. In preliminary analyses, we therefore tested whether this was fulfilled. We also explored whether posing the *Confirmation-of*-See*ing question* makes a difference to children’s TB performance. Figure 2 provides an overview of children’s performance in the various tasks (see Supplementary Material, for statistical tests of the developmental trends depicted in Figure 2 and for an analysis testing the impact of children’s gender and the order of presentation).

Data of one six-year-old is missing for the TS condition as, due to an experimental error, they received two FS trails but no TS trial.

### Preliminary Analyses

#### Comparison of True Belief and False Belief Performance

Children showed three patterns of performance: They passed both TB trials and failed at least one FB trial (*M* = 52.15 months), they passed both FB trials and failed at least one TB trial (*M* = 66.57 months), they passed both FB and TB trials (*M* = 65.17 months) (see Table 1). Overall, this yields a small to moderate negative correlation between FB and TB tasks (*Pearson’s r* = −0.28, *p* < 0.01).

**TABLE 1:** Contingency between FB and TB and FS task performance.

		TB (correct trials)		FS (correct trials)	
		0 or 1	2	0	1
FB (correct trials)	0 or 1	13 (7)	25 (11)	24	14
	2	27 (14)	23 (12)	11	39

*Numbers in parentheses indicate subset of children in the Confirmation-of-Seeing question condition.*

Asking the *Confirmation-of*-See*ing question* on TB trials before the test question had no reliable effect, *Chi-squared test* (based on a binomial distribution – children answered either both TB trials correct or not): *p* = 0.83 (see Supplementary Material for further information). Consequently, the two TB conditions (*Confirmation-of*-See*ing question*: yes/no) will be collapsed for all further analyses.

#### Comparison of False Sign and False Belief Performance

False sign and FB task performance showed a moderate to large positive correlation (*Pearson’s r* = 0.46, *p* < 0.001, see Table 1). A *McNemar* test (FB was recoded: children with two correct answers are passers, others are non-passers), revealed no significant difference in the performance of the two tasks (*p* = 0.69).

### Main Analysis

In contrast to the TB task, performance in the TS was close to ceiling: 93% of the children (*n* = 82) answered the TS test question correctly (see Table 2). A *Chi-squared test* revealed no significant difference in TS performance between children who did and did not receive the *Confirmation-of-Change question* before the test question (*p* = 0.34). TB and TS tasks were not correlated (*r* = 0.04, *p* = 0.68). A *McNemar* test (based on a binomial distribution – children either did or did not consistently pass each task), revealed a significant difference in the performance of the two tasks (*p* < 0.001). For further information, see Supplementary Material.

**TABLE 2:** Contingency between TB and TS task performance.

		TS (correct trials)		
		0	1	sum
TB (correct trials)	0 or 1	2	38	40
	2	3	44	47
	Sum	5	82	87

## General Discussion

The aim of the present study was to investigate the source of the puzzling finding that children from around age 4 begin to fail TB tasks. In particular, we tested factors that make the TB task pragmatically demanding and confusing. We compared children’s performance in the TB/FB task to performance in the TS/FS task because these tasks are closely matched in structure, involve the same kind of academic and trivial test questions, but contrast in that TB but not TS tasks implicate an alternative mental perspective or misrepresentation.

The main findings were the following: First, the pattern of negative correlations of performance in the TB and FB tasks (Oktay-Gür and Rakoczy, 2017) was replicated. Younger children tended to succeed in the TB but to fail in the FB task whereas older children tended to show the reverse pattern. Second, the convergence and correlation of FB and FS tasks were replicated. Performance in the two tasks that involve a misrepresentation is strongly correlated and develops as found in earlier studies (for an overview, see Perner and Leekam, 2008). This provides additional evidence for a joint developmental transition marking the onset of metarepresentation and perspective understanding (Perner et al., 2002, 2003, 2005; Perner and Roessler, 2012; Moll et al., 2013). Third, there was a marked dissociation between TB and TS tasks. Children showed difficulties in the TB task but not in the TS task. The critical questions in both tasks are trivial academic test questions, but in the former it suggests that there may be an alternative mental representation involved which is not the case in the latter.

Taken together, these results provide new and clear evidence that the TB test question’s reference to an agent’s rational action (that in turn evokes reference to her subjective perspective) is a crucial part of what makes the question pragmatically odd and thus difficult. In line with previous results^4^ (Oktay-Gür and Rakoczy, 2017; Rakoczy and Oktay-Gür, 2020), the present findings corroborate the assumption that it is a combination of various factors that makes TB questions particularly confusing and challenging. They are trivial academic test questions about a rational agent’s action. Their triviality and academic nature make them pragmatically odd. Importantly, this effect is particularly strong when the question refers to a rational agent’s action. We usually do not ask about a rational agent’s perspective or action unless it is unclear what the agent should do or when the agent has a deviant perspective. As this is not obviously the case in the TB question it implicates an alternative hidden mental perspective; trying to figure out what this alternative perspective may be, children venture the guess that the agent might go to the wrong location.

At the same time, the present study leaves open many fundamental questions. First, why exactly is there this sharp difference in the performance of a test question about the belief and action of an agent on the one hand, and the structurally corresponding non-mental representation of an external sign on the other hand? Do we make different kinds of rationality assumptions vis-a-vis the original intentionality of rational agents and the derived intentionality of external signs? In contrast to the intrinsic and original intentionality of agents’ mental states, the intentionality of external signs derives from the creators’ and users’ intentions that confer meaning to them (Searle, 1983). These different kinds of intentionality of mental and non-mental representations might come along with diverging rationality assumptions, and these diverging rationality assumptions may explain the diverging pattern of results in the otherwise structurally analogous tasks. Needless to say that currently this is not more than a speculation; but future studies could and should test for this possibility empirically.

Second, if indeed children find trivial academic test questions about a rational agent’s perspective and action confusing, how general is this phenomenon? Here, and in previous research we have shown it for action prediction and (true) belief ascription (Oktay-Gür and Rakoczy, 2017). But would it hold in similar ways, for the ascription of FBs and other types of mental states? For example, is a trivial academic test question about a rational agent’s desire pragmatically as confusing? Think of a scenario of the following kind: “Kate has a terrible toothache, but her dentist has the perfect drug for her that is free, has no side effects and immediately makes the pain go away. What will Kate now want to do?” Asking this question might be similarly confusing as asking the test question in the TB task. What is the point of asking about Kate’s desire in this situation in which it is completely obvious what is good and to be done? It might make us wonder “Why would someone ask me such obvious things?” and then lead us, in an attempt to make sense of the question, to try out auxiliary assumptions (“Well, perhaps she’s a masochist?”) It will be an interesting question for future studies to find out whether similar U-shaped curves, based on pragmatic confusion, can be found in mental state ascription more generally.

## Data Availability Statement

The raw data supporting the conclusions of this article will be made available by the authors, without undue reservation.

## Ethics Statement

Ethical review and approval was not required for the study on human participants in accordance with the local legislation and institutional requirements. Written informed consent for participation was not provided by the participants’ legal guardians/next of kin because the study was conducted online. In the online study, parents/legal guardians gave verbal consent before the testing was started. Verbal consent was recorded and stored separately from the recording of the test session.

## Author Contributions

LPS, MH, LMS, JP, and HR contributed to conception and design of the study. AL collected the data. LPS supervised the planning and execution process. HR provided resources for the data collection. LPS performed the statistical analysis. LPS, LMS, and AL wrote the sections and first draft of the manuscript. MH, JP, and HR gave critical review and commentary on the draft of the manuscript. All authors contributed to manuscript revision, read, and approved the submitted version.

## Conflict of Interest

The authors declare that the research was conducted in the absence of any commercial or financial relationships that could be construed as a potential conflict of interest.

## Publisher’s Note

All claims expressed in this article are solely those of the authors and do not necessarily represent those of their affiliated organizations, or those of the publisher, the editors and the reviewers. Any product that may be evaluated in this article, or claim that may be made by its manufacturer, is not guaranteed or endorsed by the publisher.

## Footnotes

1 Commonly considered knowledge that in turn implies a true belief (in contrast to TB tasks that do not imply knowledge; see Gettier, 1963).

2
https://kinderschaffenwissen.eva.mpg.de

3 For the test question in FB/TB we used this German wording: “Sag mal, wo wird Maxi denn jetzt hingehen?,” avoiding “look first.”

4 The present findings from the TB task by themselves are not strictly incompatible with alternative accounts that interpret older children’s failure in TB tasks as an indication of lacking ToM competence (Fabricius et al., 2021). According to such accounts, this pattern of results suggests that children do not use metarepresentational belief ascription until much later, but rather operate with a simple (so-called “perceptual access”) heuristic. But the results from previous studies (Oktay-Gür and Rakoczy, 2017; Huemer et al., 2019; Study 3; Rakoczy and Oktay-Gür, 2020; Studies 1 and 5) are strictly incompatible with competence limitation accounts. Taken together, these previous findings and the present results, in particular the relations found here between FB and FS tasks, thus provide comprehensive evidence for pragmatic and against competence limitation accounts.

## References

[B1] Fabricius W. V. Boyer T. W. Weimer A. A. Carroll K. (2010). True or false: do 5-year-olds understand belief? *Dev. Psychol.* 46:1402. 10.1037/a0017648 21058830

[B2] Fabricius W. V. Gonzales C. R. Pesch A. Weimer A. A. Pugliese J. Carroll K. (2021). Perceptual access reasoning (PAR) in developing a representational theory of mind. *Monogr. Soc. Res. Child. Dev.* 86, 7–154. 10.1111/mono.12432 34580875 PMC9292623

[B3] Friedman O. Griffin R. Brownell H. Winner E. (2003). Problems with the seeing = knowing rule. *Dev. Sci.* 6 505–513. 10.1111/1467-7687.00308

[B4] Gettier E. L. (1963). Is justified true belief knowledge? *Analysis* 23 121–123. 10.1093/analys/23.6.121

[B5] Hedger J. A. Fabricius W. V. (2011). True belief belies false belief: recent findings of competence in infants and limitations in 5-year-olds, and implications for theory of mind development. *Rev. Philos. Psychol.* 2 429–447. 10.1007/s13164-011-0069-9

[B6] Helming K. A. Strickland B. Jacob P. (2014). Making sense of early false-belief understanding. *Trends Cogn. Sci.* 18 167–170. 10.1016/j.tics.2014.01.005 24612994

[B7] Helming K. A. Strickland B. Jacob P. (2016). Solving the puzzle about early belief-ascription. *Mind Lang.* 31 438–469. 10.1111/mila.12114

[B8] Huemer M. Gruber S. Mangstl A. Perner J. (2019). The ‘True belief error’ in 4- to 6-year-old children. *Int. Conv. Psychol. Sci*. 10.1016/j.cognition.2022.105255 36088669

[B9] Leekam S. R. Perner J. (1991). Does the autistic child have a metarepresentational deficit? *Cognition* 40 203–218. 10.1016/0010-0277(91)90025-Y 1786675

[B10] Leekam S. Perner J. Healey L. Sewell C. (2008). False signs and the non-specificity of theory of mind: evidence that preschoolers have general difficulties in understanding representations. *Br. J. Dev. Psychol.* 26 485–497. 10.1348/026151007X260154

[B11] Moll H. Meltzoff A. N. Merzsch K. Tomasello M. (2013). Taking versus confronting visual perspectives in preschool children. *Dev. Psychol.* 49 646–654. 10.1037/a0028633 22612438

[B12] Oktay-Gür N. Rakoczy H. (2017). Children’s difficulty with true belief tasks: competence deficit or performance problem? *Cognition* 166 28–41. 10.1016/j.cognition.2017.05.002 28554083

[B13] Papafragou A. Cassidy K. Gleitman L. (2007). When we think about thinking: the acquisition of belief verbs. *Cognition* 105 125–165. 10.1016/j.cognition.2006.09.008 17094956 PMC2768311

[B14] Parkin L. J. (1994). *Children’s Understanding of Misrepresentation.* Doctoral dissertation. Brighton: University of Sussex.

[B15] Perner J. (1991). *Understanding the Representational Mind.* Cambridg, MA: The MIT Press.

[B16] Perner J. Brandl J. L. Garnham A. (2003). What is a perspective problem? Developmental issues in belief ascription and dual identity. *Facta Philos.* 5 355–378.

[B17] Perner J. Leekam S. (2008). The curious incident of the photo that was accused of being false: issues of domain specificity in development, autism, and brain imaging. *Q. J. Exp. Psychol.* 61 76–89. 10.1080/17470210701508756 18038340

[B18] Perner J. Mauer M. C. Hildenbrand M. (2011). Identity: key to children’s understanding of belief. *Science* 333 474–477. 10.1126/science.1201216 21778403

[B19] Perner J. Roessler J. (2012). From infants’ to children’s appreciation of belief. *Trends Cogn. Sci.* 16 519–525. 10.1016/j.tics.2012.08.004 22964134 PMC3460239

[B20] Perner J. Stummer S. Sprung M. Doherty M. (2002). Theory of mind finds its Piagetian perspective: why alternative naming comes with understanding belief. *Cogn. Dev.* 17 1451–1472. 10.1016/S0885-2014(02)00127-2

[B21] Perner J. Zauner P. Sprung M. (2005). “What does “that” have to do with point of view? The case of conflicting desires and “want” in German,” in *Why Language Matters for Theory of Mind*, eds Astington J. W. Baird J. A. (Oxford: Oxford University Press), 220–244.

[B22] Premack D. Woodruff G. (1978). Does the chimpanzee have a theory of mind? *Behav. Brain Sci.* 1 515–526. 10.1017/s0140525x00076512

[B23] Rakoczy H. Oktay-Gür N. (2020). Why do young children look so smart and older children look so dumb on true belief control tasks? An investigation of pragmatic performance factors. *J. Cogn. Dev.* 21 213–239. 10.1080/15248372.2019.1709467

[B24] Sabbagh M. A. Xu F. Carlson S. M. Moses L. J. Lee K. (2006). The development of executive functioning and theory of mind: a comparison of Chinese and US preschoolers. *Psychol. Sci.* 17 74–81. 10.1111/j.1467-9280.2005.01667.x 16371147 PMC2567057

[B25] Schidelko L. P. Schünemann B. Rakoczy H. Proft M. (2021). Online testing yields the same results as lab testing: a validation study with the false belief task. *Front. Psychol.* 12:4573. 10.3389/fpsyg.2021.703238 34721151 PMC8548716

[B26] Schuster B. A. Huemer M. Priewasser B. Perner J. (2021). “Preschoolers’ understanding of misrepresentation: why the false sign test overestimates performance in german-speaking children,” in *Proceedings of the Budapest CEU Conference on Cognitive Development*, Budapest.

[B27] Searle J. R. (1969). *Speech Acts: An Essay in the Philosophy of Language.* Cambridge: Cambridge University Press.

[B28] Searle J. R. (1983). *Intentionality: An Essay in the Philosophy of Mind.* Cambridge: Cambridge University Press.

[B29] Siegal M. (1999). Language and thought: the fundamental significance of conversational awareness for cognitive development. *Dev. Sci.* 2 1–14. 10.1111/1467-7687.00048

[B30] Siegal M. Beattie K. (1991). Where to look first for children’s knowledge of false beliefs. *Cognition* 38, 1–12. 10.1016/0010-0277(91)90020-5 2015753

[B31] Wellman H. Cross D. Watson J. (2001). Meta-analysis of theory-of-mind development: the truth about false belief. *Child Dev.* 72 655–684. 10.1111/1467-8624.00304 11405571

[B32] Westra E. (2016). Talking about minds: social experience, pragmatic development, and the false belief task. *Synthese* 194 4559–4581. 10.1007/s11229-016-1159-0

[B33] Wimmer H. Perner J. (1983). Beliefs about beliefs: representation and constraining function of wrong beliefs in young children’s understanding of deception. *Cognition* 13 103–128. 10.1016/0010-0277(83)90004-5 6681741

[B34] Zaitchik D. (1990). When representations conflict with reality: the preschooler’s problem with false beliefs and “false” photographs. *Cognition* 35 41–68. 10.1016/0010-0277(90)90036-J 2340712

