# Supplementary material for: Why Do Children Who Solve False Belief Tasks Begin to Find True Belief Control Tasks Difficult? A Test of Pragmatic Performance Factors in Theory of Mind Tasks
Source: Front Psychol. 2022 Jan 14;12:797246. doi: 10.3389/fpsyg.2021.797246 (PMC8796962; doi:10.3389/fpsyg.2021.797246)
Supplement: Supplementary file 1 [file Data_Sheet_1.PDF]

Supplementary Material

1 Supplementary Methods

1.1 Participants

Within the final sample, all children were native German speakers (85.2% monolingual). Eleven children were excluded from analysis because they answered ≥50% of the control questions in the belief tasks incorrectly (*n* = 9), they watched their siblings participating in the study before (*n* = 1) or participated in a very similar study shortly before (*n* = 1). Due to an error in (online) recruitment of the children, the seven most recently tested children of the respective age groups were excluded to achieve an equal distribution throughout the age groups, so that the final sample consisted of 88 children (46 female, 42 male; range = 36 – 83 months, *M* = 59.3 months), divided into groups of 3-year-olds (36 – 47 months, *M* = 42.1 months), 4-year-olds (48 – 59 months, *M* = 53.9 months), 5-year-olds (60 – 71 months, *M* = 64.5 months) and 6-year-olds (72 – 83 months, *M* = 76.7 months), each consisting of 22 children.

1.2 Design

The basic design was a mixed 2 (Confirmation-of-Seeing/Change question: yes – no) x 2 (FB/TB order: FB first – TB first) x 24 (story line order: Maxi, Lisa, Paul, Susi) factorial design resulting in 96 conditions. For the sign tasks, the order of the story line (ice-cream van – tractor) and FS/TS order (FS first – TS first) was counterbalanced across these 96 conditions. The factor *Confirmation-of-Change* question in the sign task was parallel to the Confirmation-of-Seeing question of the belief task. Children were randomly assigned to one of the conditions.

In the two belief conditions (FB/TB), we showed children two trials in each condition to get as accurate a picture as possible of the relation between FB and TB. In the two sign conditions (FS/TS), we showed the children one trial in each condition, as we were concerned that a total of eight belief and sign trials would be overwhelming for the children at this age, especially due to the online presentation of the stories.

1.3 Procedure

The child was required to give verbal answers. If children pointed at the screen instead of answering verbally, their parent was asked to name the location the child pointed at. Each session lasted approximately 30 minutes.

1.4 Belief Tasks (Example: Lisa and the teddy)

False Belief

This is Lisa's room. Tell me, what color is this box (*left box was highlighted*)? And what color is the other box (*right box was highlighted*)?
Look, here comes Lisa with her teddy. She's going to put it in the red box.
Lisa is thirsty and goes to get a drink.
Now her brother comes into the room. He is looking for something in the red box. He only finds the teddy... and he puts it in the yellow box.
Then he goes out to play.
Now Lisa comes back and wants to play with her teddy.

Test question: Where will Lisa go now? (*Correct answer: red box*)

Memory question: Where did Lisa put the teddy in the beginning? (*Correct answer: red box*)
Reality question: Where is the teddy now? (*Correct answer: yellow box*)

True Belief (Q-)

This is Lisa's room. Tell me, what color is this box (*left box was highlighted*)? And what color is the other box (*right box was highlighted*)?
Look, here comes Lisa with her teddy. She's going to put it in the red box.
Now her brother comes into the room. He is looking for something in the red box and Lisa looks what he is doing. He only finds the teddy bear... and he puts it in the yellow box. Lisa walks along and watches him.
Then he goes out to play.
Lisa is thirsty and goes to get a drink.
Now Lisa comes back and wants to play with her teddy.

Test question: Where will Lisa go now? (*Correct answer: yellow box*)
Memory question: Where did Lisa put the teddy in the beginning? (*Correct answer: red box*)
Reality question: Where is the teddy now? (*Correct answer: yellow box*)

True Belief (Q+)

This is Lisa's room. Tell me, what color is this box (*left box was highlighted*)? And what color is the other box (*right box was highlighted*)?
Look, here comes Lisa with her teddy. She's going to put it in the red box.
Now her brother comes into the room. He is looking for something in the red box and Lisa looks what he is doing. He only finds the teddy bear... and he puts it in the yellow box. Lisa walks along and watches him.
Then he goes out to play.

Confirmation-of-Seeing question Q+ (TB): Did Lisa see her brother put the teddy in the yellow box? (*Correct answer: yes*)

Lisa is thirsty and goes to get a drink.
Now Lisa comes back and wants to play with her teddy.

Test question: Where will Lisa go now? (*Correct answer: yellow box*)
Memory question: Where did Lisa put the teddy in the beginning? (*Correct answer: red box*)
Reality question: Where is the teddy now? (*Correct answer: yellow box*)

1.5 Sign Tasks (Example: Ice cream van)

The sign task (adapted from Parkin, 1994) was presented as video clips animated with the animation software *Vyond Studio* (GoAnimate, 2017) in two storylines.

Familiarization

Look, here is a crossroads. Do you see it? On one side you see a church. And on the other side is a farm.
Sometimes an ice cream van drives along the road. Look, there it is! It stops then either behind the church or behind the farm.
If there is a blue sign at the intersection, then the ice cream truck is behind the farm.
If there is a green sign on the intersection, then the ice cream truck is behind the church.
So the sign always tells where the ice cream truck is.

Familiarization question 1: Tell me, if the sign is blue, where is the ice cream truck? (*Correct answer: farm*)

If correct: That's right, if the sign is blue, the ice cream van is behind the farm.
If wrong: We just saw that the ice cream van is behind the farm when the sign is blue. So where is the ice cream van when the sign is blue? – Right, when the sign is blue, the ice cream van is behind the farm.

Familiarization question 2: And if the sign is green, where is the ice cream van? (*Correct answer: church*)

If correct: That's right, if the sign is green, the ice cream van is behind the church.
If wrong: We just saw that the ice cream van is behind the church when the sign is green. So where is the ice cream van when the sign is green? – Right, when the sign is green, the ice cream van is behind the church.

False Sign

Look, here comes the ice cream van again. It turns off... and stops behind the church.
Now it's moving again.
Look, the ice cream van has stopped. Look, the ice cream van is moving on and the sign remains as it is. Then the ice cream van moves on...
...and stops behind the farm.

Test question: What does the sign say where the ice cream van is? (*Correct answer: church*)
Reality question: Where is the ice cream van now? (*Correct answer: farm*)
Memory question: And where was it right before? (*Correct answer: church*)

True Sign (Q-)

Look, here comes the ice cream van again. It turns off... and stops behind the church.
Now it's moving again.
Look, the ice cream van has stopped. Now the sign has been changed. Then the ice cream van moves on...
...and stops behind the farm.

Test question: What does the sign say where the ice cream van is? (*Correct answer: farm*)
Reality question: Where is the ice cream van now? (*Correct answer: farm*)
Memory question: And where was it right before? (*Correct answer: church*)

True Sign (Q+)

Look, here comes the ice cream van again. It turns off... and stops behind the church.
Now it's moving again.
Look, the ice cream van has stopped. Now the sign has been changed. Then the ice cream van moves on...
...and stops behind the farm.

Confirmation-of-Change question Q+ (TS): Has the sign been changed? (*Correct answer: yes*)

Test question: What does the sign say where the ice cream van is? (*Correct answer: farm*)
Reality question: Where is the ice cream van now? (*Correct answer: farm*)
Memory question: And where was it right before? (*Correct answer: church*)

1.6 Coding

The participants' answers were coded by the experimenter. Correct answers (*see* Procedure) were coded with 1. Every answer that deviated from the correct answers were coded as 0. A second coder, blind to the hypotheses of the present study, coded the answers to the test questions of 20% of the videos for interrater reliability showing substantial to perfect agreement of the raters (Cohen's κs ≥ .89).

2 Supplementary Results

2.1 Testing the impact of children's age, gender and the order of tasks

We conducted additional analyses to test for the impact of children's age, gender, the task type (true vs. false) and the order of presentation (true version first vs. false version first) on the performance in the belief and the sign task (for descriptive data, *see* Fig S1).

To this end, we set up a first linear regression control model for the belief task including the main effects of children's age in months, children's gender, presentation order and belief type and interactions. For the belief task, we would expect an interaction between age and belief type: Children's performance on the FB task increases with age while it decreases for the TB task. Additionally, we test for an interaction of the presentation order with the belief type.

> Belief Model 1: Number of correct belief trials ~ age + gender + presentation order + belief type + age x belief type + presentation order x belief type

This model showed a significant main effect of belief type (B = - 2.43 , $p < .001$) and significant interactions between age and belief type (B = 0.05, $p < .001$) and between belief type and presentation order (B = - 0.68, $p < .01$). The model showed no main effect of children's age (B = -0.01, p = .10), children's gender (B = -0.09, p = .45) and presentation order (B = .25, p =.16).

As gender had no impact, we conducted a second, main model excluding gender.

> Belief Model 2: Number of correct belief trials ~ age + presentation order + belief type + age x belief type + presentation order x belief type

The comparison of Model 1 (Adj. R^2= .16) and Model 2 (Adj. R^2= .16) was not significant (F= 0.57, p = .45) as gender had no impact. The detailed results of main belief model 2 are reported in Table S1.

Table S1

Results of the full regression model on the impact of children's age, belief type and presentation order in the FB and TB task

	B	*SE B*	*p*
Full Model			
Intercept	1.77	0.41	< .001***
Age in months	- 0.01	0.01	.11
Belief type	- 2.43	0.58	< .001***
Presentation order	0.25	0.18	.15
Age * Belief Type	0.05	0.01	< .001***
Presentation order * Belief Type	- 0.68	0.25	< .01**

Note. Belief type: False or True Belief, Presentation order: block of FB or TB tasks first.

In accordance with the literature, children's performance in the FB task increased and decreased in the TB task with age. As post-hoc test, we computed two-sided Wilcoxon signed rank tests against chance level performance (= 1.0) for the four age groups in the two belief conditions. For results, *see* Table S2.

Regarding presentation order, children's performance in the belief tasks varied depending on the order in which they were presented. The performance for both FB and TB tasks was higher when presented as the first block compared to when presented as the second block. This effect was stronger for the false belief trials. Figure S1 shows the number of children passing the first and second TB and FB trial dependent of the presentation order. A similar effect was found previously in Rakoczy and Oktay-Gür (2020, *Exp. 5*).

Table S2

Results of Wilcoxon signed rank test against chance level performance (= 1.0) for the age groups in the FB and TB task

	False Belief Task			True Belief task		
	M	*p*	*r*	*M*	*p*	*r*
3-year-olds	0.45	< .01**	-0.60	1.59	< .01**	-0.67
4-year-olds	1.45	< .05*	-0.47	1.14	.48	-0.15
5-year-olds	1.27	.19	-0.28	1.18	.38	-0.19
6-year-olds	1.77	< .001***	-0.79	1.09	.67	-0.09

Figure S1

Number of children passing and failing the first and second trial of the TB and FB task across age groups

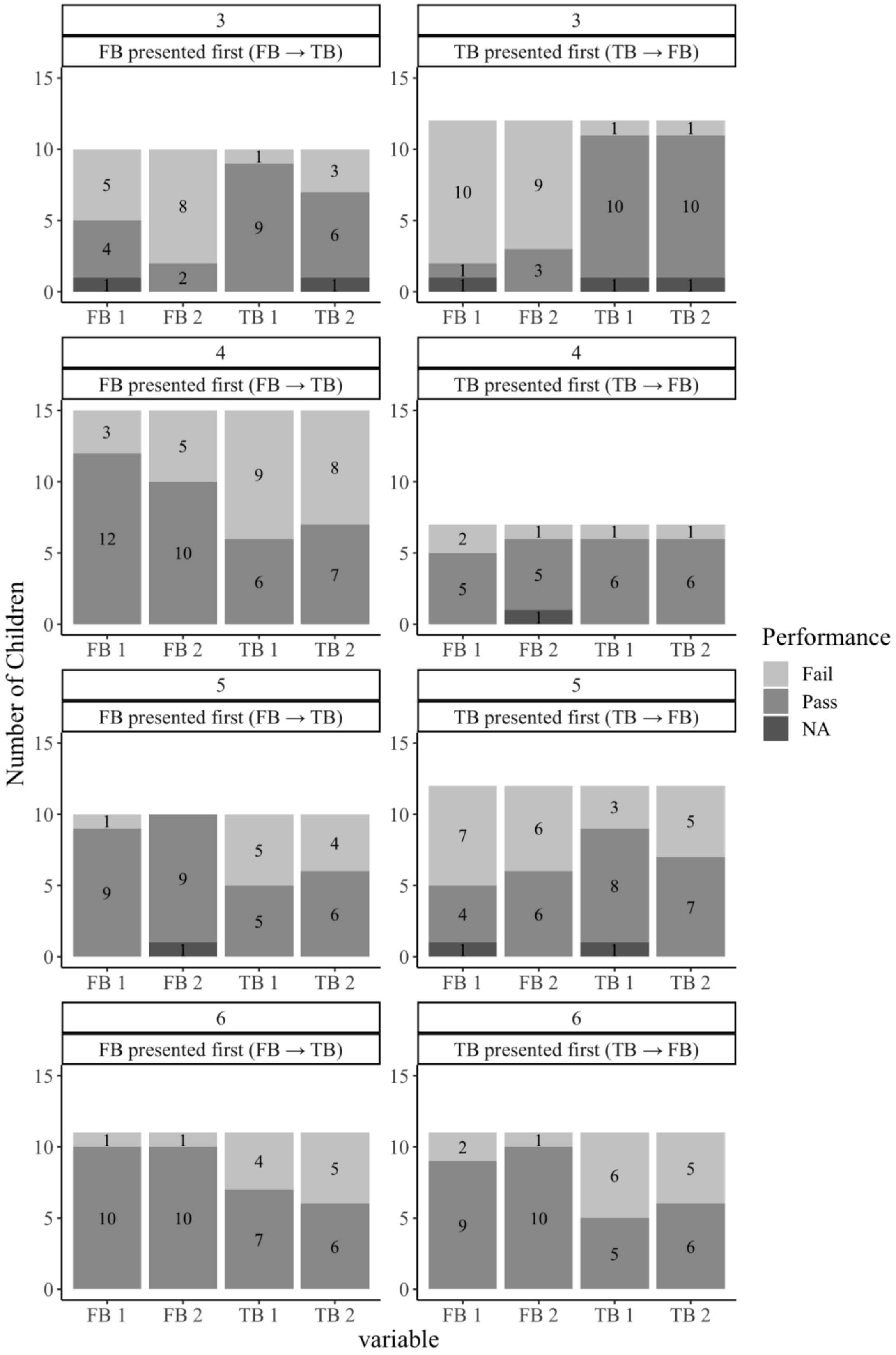

Note. FB 1 and TB 1 indicates first trials of False and True Belief Task, FB 2 and TB 2 indicates second trials of False and True Belief Task.

We used the same approach to test for the impact of children's age, gender, the sign type and the presentation order on the sign task. We set up a first logistic regression control model in which we included the main effects of children's age in months, children's gender, sign type and presentation order and the interaction effects. For the sign task, we expect an interaction between age and the sign type: Children's performance on the FS task increases with age while it is constantly high in the TS task. Additionally, we test for an interaction of the presentation order and sign type.

> Sign Model 1: number of correct sign trials ~ age + gender + sign type + presentation order + age x sign type + presentation order x sign type

This model shows a significant main effect of the sign type (B = -7.83, p <.01) and a significant interaction effect of age and sign type (B = 0.10, p < .05). The model shows no main effect of children's age (B = 0.00, p =.92), gender (B = 0.51, p =.28) or presentation order (B = 1.40, p =.22), nor an interaction effect of sign type and presentation order (B = -1.48, p =.24).

As gender had no impact, we conducted a second, main model excluding gender.

> Sign Model 2: Sign trial: correct – incorrect ~ age * sign type + presentation order * sign type

The full-null model comparison was not significant ($X^2(1) = 1.20$, p = .27) as gender had no impact. The detailed results of main sign model 2 are reported in Table S3.

Table S3

Results of the logistic regression model on the impact of children's age, sign type and presentation order in the FS and TS task

	B(*SE*)	*z*	*p*	95% CI for Odds Ratio		
				Lower	Odds Ratio	Upper
Included						
Age in months	0.00	0.02	.99	0.93	1.00	1.08
Sign type	-7.83	-3.12	< .01**	0.00	0.00	0.05
Presentation order	1.43	1.26	.21	0.59	4.20	84.06
Age * Sign Type	0.10	2.46	< .05*	1.02	1.11	1.21
Presentation order * Sign type	-1.48	-1.19	.24	0.01	0.23	2.09

Note. R^2= .60 (Nagelkerke).

As post-hoc test, we computed two-sided Wilcoxon signed rank tests against chance level performance (=0.5) for the four age groups in the two sign conditions. For results, *see* Table S4.

Table S4

Results of Wilcoxon signed rank test against chance level performance (0.5) for the age groups in the True and False Sign task

	False Sign Task			True Sign task		
	M	*p*	*r*	*M*	*p*	*r*
3-year-olds	0.18	< .001***	-0.63	0.91	< .001***	-0.81
4-year-olds	0.64	< .001***	-0.27	0.95	< .001***	-0.91
5-year-olds	0.73	< .001***	-0.45	1	< .001***	-1.00
6-year-olds	0.86	< .001***	-0.72	0.90	< .001***	-0.81

In accordance with our predictions, children’s FS performance increased with age while TS performance was close to or at ceiling across all age groups. The presentation order did not have an impact on performance. Figure S2 shows the number of children passing the true and false sign trial dependent of the presentation order.

Figure S2

Number of children passing and failing the FS and TS task across age groups

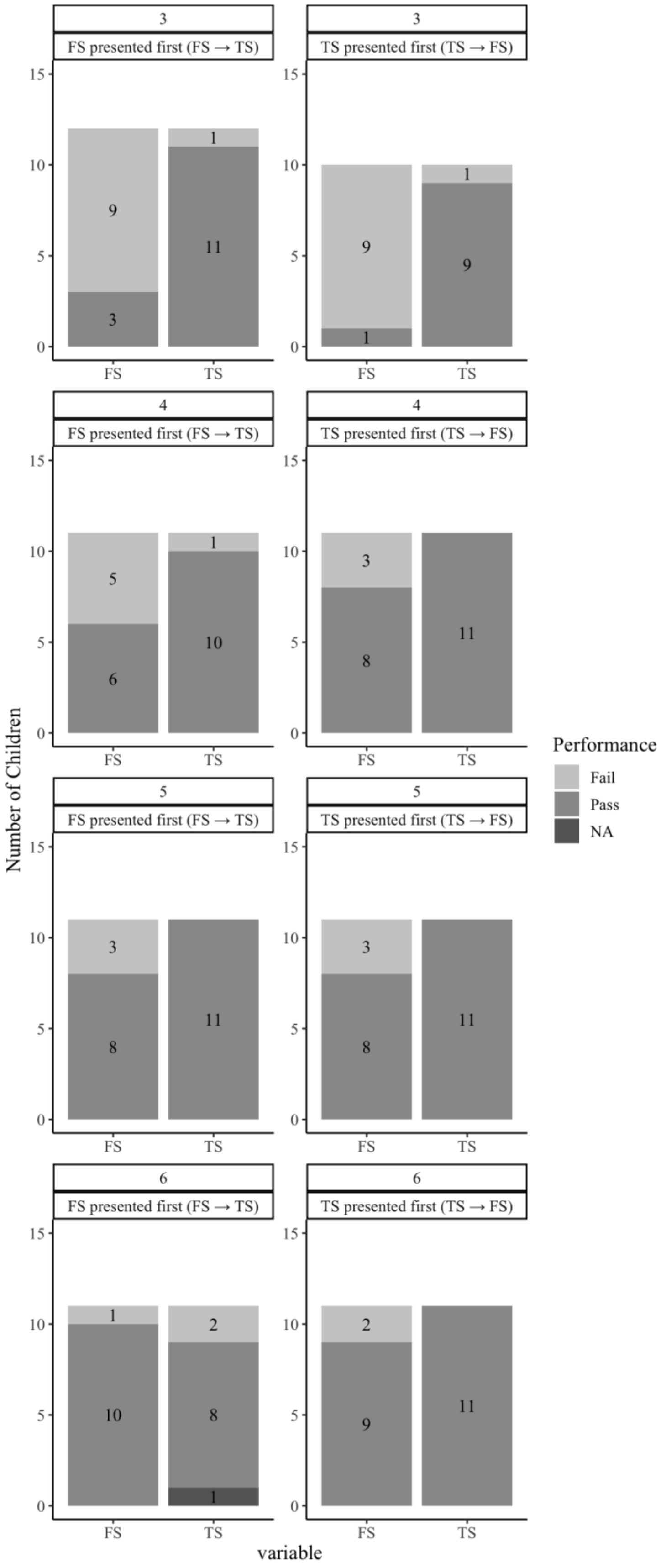

2.2 Performance in Confirmation-of-Seeing question (Q+) and Confirmation-of-Change question (Q+)

Half of the children (*n* = 44) received the Confirmation-of-Seeing question (Q+) in the TB trials and the Confirmation-of-Change question in the TS trial. 37 of these children answered the confirmation-of-seeing question in both the first and second TB trial correctly ("yes"). 23 of the 37 children answered the respective TB test question correctly.

35 of the 44 children answered the Confirmation-of-Change question correctly ("yes"). The majority of children who did not answer the confirmation-of-change question with "yes" did not answer the question with "no" but answered directly with the color of the sign ("red") or the location of the vehicle ("farm"). All of the 35 children answered the respective TS test question correctly.

2.3. Analyses with first trial only

2.3.1. Comparison of TB and FB performance with first trial only

The performance in the first TB trial and first FB trial showed a small to moderate negative correlation between FB and TB tasks (*Pearson's* $r = -.33$, $p < .01$).

2.3.2. Comparison of FS and FB performance with first trial only

FS and FB task performance showed a moderate to large positive correlation (*Pearson's* r = .50, $p < .001$). A *McNemar* test revealed no significant difference in the performance of the two tasks ($p = .82$).

2.3.3 Main analysis with first trial only

The performance in the first trial of the TB task and TS task were not correlated ($r = .02$, $p = .82$). A *McNemar* test revealed a significant difference in the performance of the two tasks ($p < .001$).

References

GoAnimate (2017). *Vyond* [Software]. https://www.vyond.com/
